# The Perceptions of Medical School Students and Faculty Toward Obesity Medicine Education: Survey and Needs Analysis

**DOI:** 10.2196/mededu.7361

**Published:** 2017-11-09

**Authors:** Mary Metcalf, Karen Rossie, Katie Stokes, Bradley Tanner

**Affiliations:** ^1^ Clinical Tools, Inc. Chapel Hill, NC United States

**Keywords:** obesity, weight loss, medical students, medical education, curriculum

## Abstract

**Background:**

Recent trends in obesity show that over two-thirds of US adults are considered at least overweight (body mass index, BMI≥25 kg/m^2^) and of those, about one-third are categorized as obese (BMI≥30 kg/m^2^). Physicians can address the health impacts of obesity; yet research has suggested that physicians-in-training frequently fail to recognize obesity, are not properly educated regarding treatment options, and spend relatively little clinic time treating obesity. Medical school is a unique opportunity to address this area of need so that the doctors of tomorrow are prepared to treat obesity appropriately.

**Objectives:**

The objective of this study was to determine perceptions of where clinical training for medical students on the topic of obesity and its treatment should improve and expand so that we could address the needs identified in a computerized clinical simulation.

**Methods:**

We conducted a literature review, as well as a needs analysis with medical school students (N=17) and faculty (N=12). Literature review provided an overview of the current state of the field. Students provided input on their current needs, learning preferences, and opinions. Faculty provided feedback on current training and their perceptions of future needs.

**Results:**

Most students were familiar with obesity medicine from various courses where obesity medicine was a subtopic, most frequently in Biochemistry or Nutrition, Endocrinology, and Wellness courses. Student knowledge about basic skills, such as measuring waist circumference, varied widely. About half of the students did not feel knowledgeable about recommending weight loss treatments. Most students did not feel prepared to provide interventions for patients in various categories of overweight/obesity, patients with psychosocial issues, obesity-related comorbidities, or failed weight loss attempts. However, most students did feel that it was their role as health professionals to provide these interventions. Faculty rated the following topics as most important to supplement the curriculum: patient-centered treatment of weight, bringing up the topic of weight, discussing weight and well-being, discussing the relationship between weight and comorbidities, and physician role with overweight or obese patients.

**Conclusions:**

A review of the literature as well as surveyed medical students and faculty identified a need for supplementation of the current obesity medicine curriculum in medical schools. Specific needed topics and skills were identified.

## Introduction

### Extent of the Public Health Problem

The consequences of obesity are broad and severe, and a majority of the population is affected. Two-thirds of US adults are considered at least overweight (body mass index, BMI≥25 kg/m^2^), and further categorization of that group shows that around one-third of adults are categorized as obese (BMI≥30 kg/m^2^) [1,2]. Obesity is the second leading cause of preventable death in the United States [3,4]. As the rate of obesity continues to rise, so does the rate of obesity’s common comorbidities. For example, according to the Centers for Disease Control and Prevention, the rate of diagnosed diabetes mellitus has more than doubled in the past 24 years, and an estimated 90% to 95% of individuals with diabetes have type 2 diabetes [5]. Patients who are obese are also at increased risk for many other chronic diseases, including hypertension, heart disease, and cancer, as well as physical disabilities [6]. Direct costs for obesity-related illnesses in the United States have reached US $147 billion/year, of which 40% can be attributed to the 8% of patients who are severely obese (BMI≥35 kg/m^2^) [6,7].

### The Gap in Between Ideal Obesity Medicine and Actual Practice

The appropriate practice of obesity medicine could turn this epidemic around. However, some evidence shows a gap between the ideal scenario and what is actually happening in practice. Providing weight management tailored for each patient, including making appropriate referrals for comprehensive care, can be effective in producing lasting weight loss [7]. Unfortunately, few physicians routinely provide screening or intervention for weight problems for their patients [7,8]. Research has suggested that physicians-in-training frequently fail to recognize obesity, are not properly educated regarding treatment options, and spend relatively little clinic time treating obesity [9]. Some students also hold negative attitudes toward people who are obese or feel uncomfortable discussing the topic, which could interfere with effective treatment. There is a gap between ideal obesity medicine and actual practice that needs to be addressed.

### Current Obesity Medicine Training in Medical Schools

It is widely recognized that medical students need to be adequately prepared to practice obesity medicine effectively. The Association of American Medical College (AAMC) recognizes the universal importance of weight management, including the prevention of overweight and obesity, and its guiding principles recommend that this be emphasized in the medical school curriculum [10]. Furthermore, the AAMC has concluded that, in addition to the disease-centered approach often taken, medical students need “easily accessible tools to focus students’ and trainees’ attention on urgent social and behavioral dimensions of patients’ complex problems, such as obesity” [11].

Despite these recommendations, research shows that obesity is often neglected in the medical school curriculum. Survey results indicate that the average American medical school spends only 19.6 of the required 25 contact hours on nutrition instruction, and only 27% of medical schools meet AAMC curriculum recommendations [7,12]. Despite known negative bias by some medical students toward obese patients [7,13-15], little is being done to change student attitudes [4]. In a meta-analysis of literature about educating medical students on obesity, Vitolins et al found only 5 publications between 1966 and 2010 describing and evaluating educational interventions [4]. It is therefore not surprising that a majority of physicians surveyed in 2 studies reported a lack of training and competence in weight management [7,8].

Medical students who are better prepared to approach the subject of obesity may improve patient care as physicians and impact patient health positively through more effective screening, diagnosis, and treatment of obesity. More effective treatments will also reduce the prevalence of obesity-related comorbid conditions such as cardiovascular disease and type 2 diabetes. Regrettably, obesity is not adequately covered in many medical schools [6,7,12]. Medical students, residents, and practicing physicians need more training about obesity and treatment options. The current medical curriculum on obesity medicine appears to have deficits that need to be clearly identified so that medical student training in this area can be improved.

To develop an understanding of the specific needs for training in obesity management skills that could be addressed via a simulation application, we conducted a needs analysis with medical school students and faculty.

## Methods

A thorough literature review was conducted, which identified possible topics for the skills training activity and common barriers that we should strive to overcome. The medical student survey responses provided an understanding of the medical students’ perceived needs and the skills they should be taught related to obesity medicine and treating patients with obesity. Faculty at different institutions gave their opinions related to medical student training in obesity medicine.

### Literature Review Methods

Before the needs analysis, we conducted a thorough literature review of the following terms in combinations to form search criteria and searched via Google Scholar, UNC Library, and PubMed: medical school, obesity, obesity medicine, education, curriculum, training, weight, and nutrition. These Web-based searches were filtered to access original research and review studies with abstracts dating from 2011 to 2015. A total of 35 study abstracts were reviewed for relevance, and 11 studies were selected for further reading. We collated topics and skills that had been identified in these studies as needing improvement or additional training, as well as common barriers to successful skills training. Our needs analysis survey for medical school faculty was designed to inform us of the subtopics and skills related to obesity medicine that were most needed to supplement medical school curricula.

### Medical Student and Faculty Survey Methods

To balance the literature review, we conducted a short survey of current medical students and another of medical school faculty members. For this study, a convenience sample was used. Recruitment emails were sent to medical students who had previously taken a health professional student course provided by Clinical Tools, Inc. Recruitment was targeted at 2nd, 3rd, and 4th year medical school students, 1st year residents, and medical interns. Participants came from 4 medical schools and included 11 female and 6 male students. Out of the 17 medical students, 9 (53%) were 2nd year students, 7 (41%) were 3rd year students, and 1 (6%) was in the 4th year of medical school training. All major medical specialties were indicated as possible areas of interest for the students.

Faculty participants were course or clerkship directors, professors, or coordinators in Internal Medicine, Family Medicine, or Medical Biochemistry departments. Faculty emails were obtained from Web-based searches of reputable medical schools in the areas mentioned above. For a group total of 12 faculty, 7 participants were female and 5 were male.

Both groups were emailed a link to a screening and eligibility form. After completing the Web-based eligibility form, only eligible users were directed to complete the needs analysis survey via the same link.

The survey questions were presented in several formats, including Likert-style items, multiple-choice questions, and open-ended questions. The survey took approximately 15 min to complete.

The medical student survey questions were broken down into the following categories:

1. Obesity medicine: Survey questions in this section assessed students’ familiarity with obesity medicine in their medical school and what courses on obesity medicine were included.
2. Medical education on obesity: In this set of questions, students provided feedback about their current training in topics related to assessing and treating overweight and obesity. In addition, they rated their knowledge or preparation and how they perceived their roles in providing interventions for patients with overweight or obesity.
3. Further information and thoughts: Students were asked to provide additional feedback about what they viewed as the most challenging aspect of treating overweight and obese patients.

Faculty were asked specifically about the need for training on obesity, barriers to such training, and the specific skills that should be taught, such as interviewing, motivating patients, documenting weight, assessing for appropriateness of medication or surgical intervention, developing a weight management plan, and implementing that plan.

The survey questions were broken down into the following categories:

1. Obesity medicine and curriculum: Questions were designed to assess how obesity medicine was currently taught in medical school and to assess faculty confidence in student clinical skills as they related to assessing and treating patients with overweight or obesity.
2. Topics for supplemental educational outcomes: Questions rated the importance of topics for our curriculum. Questions were divided into (1) core concepts and patient data collection, (2) patient assessment, (3) treatment approaches, (4) treatment implementation, and (5) additional topics.
3. Supplemental resource use: Questions assessed how the planned educational simulation would be integrated into the medical school curriculum in terms of where within the curriculum it would be included, how it would be used, time spent, and its usefulness.

## Results

### Literature Review Results

Through the various studies, gaps were identified in our Web-based literature review, which indicated a need for the following:

- Primary care providers to improve efficacy in helping patients lose weight [16,17]
- A stronger physician background in the biologic and pathophysiological foundations of obesity [9,18]
- Navigating the complexity and heterogeneity of overweight and obesity [18]
- Overcoming attitudes and bias toward patients with obesity and positive attitudes toward the actual management of obesity [4,19]
- Recognition of modest weight reduction as substantial (for comorbidity prevention)
- Access to resources
- Effective role models in a clinical setting as well as engagement in training [6]
- Training regarding obesity counseling [20]

### Student Survey Results

#### Student Survey: Obesity Medicine Courses

We asked students how obesity medicine was taught in their medical schools currently (Table 1), whether in its own course, within another course, or not at all. Of the respondents, 76% (13/17) were familiar with obesity medicine through a course where obesity medicine was a topic. Students reported obesity medicine being covered in multiple courses (Table 2), most frequently Biochemistry or Nutrition (94%; 16/17) and Endocrinology (82%; 14/17).

**Table 1:** Students’ descriptions of their obesity medicine training (N=17).

Description		n (%)
**I am familiar with obesity medicine through:**		
	A course where obesity medicine was a topic.	13 (76)
	A specific course on obesity medicine.	2 (12)
I am not familiar with obesity medicine in my coursework yet.		2 (12)

**Table 2:** System or disease topics associated with obesity medicine (N=17).

System or disease topics		n (%)
**Obesity medicine is included with these system or disease topics (choose as many as apply):**		
	Biochemistry or Nutrition	16 (94)
	Endocrinology	14 (82)
	Wellness	12 (70)
	Cardiovascular	11 (65)
	Clinical Foundations of Medicine	5 (29)

#### Student Survey: Current Training

Students were asked whether they had received training in particular topics related to assessing and treating overweight or obese patients. A majority agreed or strongly agreed that they had studied or received training to:

- Assess overweight or obese patients (65%; 11/17)
- Use behavioral counseling in clinical interviews (71%; 12/17)
- Recommend treatment for overweight or obesity (65%; 11/17)

Students agreed least often that they had studied or received training on “monitoring overweight or obesity treatment” (53%; 9/17).

#### Student Survey: Knowledge/Preparation

Students were asked to rate their knowledge about measuring and interpreting body mass assessments and treatments for overweight or obesity (Table 3). Students’ knowledge varied with the topic. The topics where students most frequently rated their knowledge as good or excellent were “calculating BMI” (82%, 14/17) and “interpreting BMI” (71%, 12/17). The topics for which students most frequently rated their knowledge the lowest (very poor or poor) were “interpreting waist circumference” (35%, 6/17) and “measuring waist circumference” (35%, 6/17).

#### Student Survey: Self-Efficacy

Students were asked how prepared they felt for interventions with patients having various stages of obesity and obesity-related problems (Table 4). A majority of students felt they were not prepared or only somewhat prepared for providing weight-related interventions to all categories of patients surveyed. On a 4-point Likert-type scale, 88% (14/16) rated their preparation the lowest for providing interventions to overweight to stage III obesity (not prepared at all or somewhat prepared), and 69% (11/16) rated low preparation for providing interventions to patients with psychosocial issues (not prepared at all or somewhat prepared). A not applicable (N/A) option was available for students to choose if they were not aware of interventions at the various stages of obesity. N/A results were not included in the question totals as noted in Table 4.

#### Student Survey: Future Role as a Health Professional

Students were asked about how they viewed their future roles in obesity management. All students (N=17) agreed or strongly agreed that they see the following as their role as a health professional:

- Recommending dietary changes
- Recommending physical activity changes
- Assessing for weight-related comorbidities

Students mostly agreed or strongly agreed that their other roles in obesity management as a health professional included:

- Selecting patients for surgical treatment (82%, 14/17)
- Selecting patients for use of pharmacotherapy (82%, 14/17)
- Providing behavioral counseling (76%, 13/17)

#### Student Survey: Experience With Overweight or Obese Patients

Students were asked a conditional question about whether they had any experience with overweight or obese patients in their case studies or clinical experiences (N=17). Additionally, when they responded that weight was addressed, they were asked about the nature of the interaction, and 71% (11/17) of the respondents said they have had adult patient encounters in which weight was addressed and the data suggested the following:

Many or most interactions involved:

- Weight as a contributing factor to a current medical condition (67%, 7/11)
- Discussions of weight initiated by the provider (45%, 5/11)
- A current medical condition exacerbated by weight problems (55%, 6/11)

Few or some interactions involved:

- Discussions of weight initiated by the patients (91%, 10/11)
- Weight as the primary focus of the appointment (91%, 10/11)
- Discussions of weight initiated by the provider (55%, 6/11)

Additionally, 59% (10/17) reported that they have encountered overweight or obese patients (or case studies) where they thought weight should have been addressed and it was not. When asked to select the reason why they thought weight was not addressed:

- 70% (7/10) selected a lack of time to discuss weight problems
- 60% (6/10) said there were more important items to discuss in the situation
- 30% (3/10) said the patient had a history of not following lifestyle change advice

**Table 3:** Students’ rating of obesity medicine knowledge (N=17).

Student knowledge		Mean (standard deviation)	Good or excellent, n (%)
**Please rate your knowledge about the following:**			
	Calculating body mass index (BMI)	4.18 (0.728)	14 (82)
	Interpreting BMI	3.88 (0.857)	12 (71)
	Recommending appropriate weight loss treatments	3.24 (0.903)	8 (47)
	Measuring waist circumference as an obesity assessment	3.18 (1.24)	6 (35)
	Interpreting waist circumference as an obesity assessment	3 (1.06)	4 (24)
Average		3.49 (0.957)	8.8 (52)

**Table 4:** Students’ rating of their obesity medicine self-efficacy (N=17).

Student self-efficacy		Mean (standard deviation)	Prepared or very prepared, n (%)
**I feel prepared for interventions with the following adult populations:**			
	Patients with obesity-related comorbidities	2.47 (0.717)	8 (47)
	Patients with psychosocial issues	1.94 (0.929)	5 (31)^a^
	Overweight or obese patients with a history of failed weight loss attempts	1.76 (0.970)	4 (24)
	Overweight to stage III obese patients	1.88 (0.619)	2 (12)^a^

^a^n=16.

#### Student Survey: Patient Communication

In round 1 of the survey, students were asked whether they were comfortable discussing weight issues with overweight or obese patients. Of the respondents, students agreed or strongly agreed they were slightly more comfortable discussing weight with obese patients (57%, 4/7) than with overweight patients (43%, 3/7).

In round 2 of the survey, students were asked about their interest in learning more about effective ways to address weight-related issues with overweight or obese patients. Of the 10 respondents, most students agreed or strongly agreed that they were interested in:

- Learning how to effectively address weight issues with obese patients (100%, 10/10)
- Learning how to effectively address weight issues alongside issues perceived as more important (90%, 9/10)
- Learning how to effectively address weight issues with overweight patients (80%, 8/10)

### Faculty Survey

#### Faculty Description of Obesity Medicine Topics Currently Covered

Obesity medicine was most often incorporated into courses on other topics according to a majority of the 12 faculty respondents (83%, 10/12) as opposed to a course focused on only obesity (8%, 1/12) or not being included in the curriculum (8%, 1/12). From the 12 universities represented, courses that faculty reported covering obesity most often were as follows: Endocrinology (58%, 7/12), Cardiovascular (50%, 6/12), and Biochemistry and/or Nutrition (42%, 5/12). Other courses covering obesity were Wellness, Gastrointestinal, and Family Medicine and Psychiatry clerkships.

BMI and physical activity guidelines were the most frequently covered of the curriculum subtopics surveyed, with 83% (10/12) of faculty agreeing or strongly agreeing. The least commonly covered subtopic was motivational counseling or interviewing techniques; only 67% (8/12) of faculty agreed or strongly agreed that this topic was included in their school’s standard curriculum.

#### Faculty Perception of Importance of Supplemental Obesity Medicine Topics

Faculty rated a list of 27 topics on their importance for inclusion in a curriculum supplement. The topics that they rated highest, using a 5-point Likert-type scale, were as follows: “patient-centered treatment of weight” (4.7), “bringing up the topic of weight” (4.6), “discussing weight and well-being” (4.6), “discussing the relationship between weight and comorbidities” (4.6), “physician role with overweight or obese patients” (4.6), “confronting personal bias against overweight or obese patients” (4.5), “referring patients for dietary guidance” (4.5), and “developing a long-term plan” (4.5).

Topics rated the lowest were “effectiveness of weight-loss surgery” (4.0), “assessing body mass” (3.9), “using the body mass assessment to guide treatment” (3.9), “referring patients for surgery” (3.8), and “personal weight struggles” (3.4).

#### Faculty Assessment of Student Clinical Skills

Faculty participants reported confidence in student skills related to evaluation and assessment of patient weight or obesity but less confidence in student ability to recommend weight loss treatments or treatment planning for weight loss (Table 5). In contrast, of the majority of faculty respondents who were aware of their students’ preparedness, 100% (8/8) rated students as only “somewhat prepared” or “not prepared at all” to follow guidelines for treating obese patients or for overweight or obese patients who had tried and failed to lose weight (Table 6).

Faculty confidence in their students’ clinical skills in obesity medicine varied according to the specific skill. For each of these questions, there was an N/A answer available; *I don’t know* in Table 5 and *Unsure* in Table 6. N/A results are not included in the question totals, varying the N=12 and given no weight in the data table. We found that faculty agreed or strongly agreed with the following:

Faculty were *confident* in their students’ ability to (see Table 5):

- Evaluate patient with overweight or obesity (64%, 7/11)
- Assess patients with overweight or obesity (82%, 9/11)

Faculty were less confident in their students’ ability to (see Table 5):

- Recommend appropriate weight loss treatments (18%, 2/11)
- Develop a long-term plan for patient weight loss (18%, 2/11)
- Implement a long-term plan for patient weight loss (10%, 1/10)

Faculty rating of student preparedness also varied with the patient category:

Faculty rated students as *not prepared* or only *somewhat* prepared to follow weight loss guidelines for treating (see Table 6):

- Overweight patients (73%, 8/11)
- Stage I-II obese patients (67%, 6/9)
- Stage III obese patients (89%, 8/9)
- Patients with psychological issues (100%, 10/10)
- Overweight or obese patients with a history of failed weight loss attempts (100%, 8/8)

Faculty rated student preparation to treat patients having weight-related comorbidities using weight loss guidelines as moderate: 55% (6/11) of faculty rated students as *prepared*, and 45% (5/11) of faculty rated students as only *somewhat prepared*.

#### Faculty Survey: Key Findings Summary

Most of the faculty reported that obesity medicine is covered within more than one course, most often Endocrinology, Cardiology, and Biochemistry or Nutrition. For obesity medicine topics, a majority of the faculty agreed or strongly agreed that body mass evaluation, dietary guidelines, and physical activity guidelines were already covered in their curriculum. Many topics were identified as important for inclusion in a supplement to the existing curriculum, with the highest rated of these topics in terms of importance involving the doctor-patient relationship and counseling the patient. Faculty were most confident in their students’ weight assessment skills and least confident in students’ ability to develop a long-range weight loss plan. Faculty rated student preparedness highest for following guidelines with patients having weight-related comorbidities and overweight patients and lowest for treating patients having failed weight loss attempts.

**Table 5:** Faculty’s assessment of students’ confidence (N=12).

Confidence in students’ skills^a^		I don't know, n (%)	Strongly disagree, n (%)	Disagree, n (%)	Neither disagree nor agree, n (%)	Agree, n (%)	Strongly agree, n (%)	Mean (SD)
**I am confident in my matriculating students' clinical skills to:**								
	Evaluate patients with overweight or obesity	1 (−^b^)	0 (0)	0 (0)	4 (36)	6 (55)	1 (9)	3.72 (0.647)
	Assess patients with overweight or obesity	1 (−)	0 (0)	0 (0)	2 (18)	7 (64)	2 (18)	4 (0.632)
	Recommend appropriate weight loss treatments	1 (−)	0 (0)	4 (36)	5 (45)	2 (18)	0 (0)	2.82 (0.751)
	Develop a long-term plan for patient weight loss	1 (−)	1 (9)	3 (27)	5 (45)	2 (18)	0 (0)	2.73 (0.905)
	Implement a long-term plan for patient weight loss	2 (−)	1 (10)	3 (30)	5 (50)	1 (10)	0 (0)	2.6 (0.843)

^a^Likert rating: I don’t know=N/A; Strongly disagree=1; Disagree=2; Neither disagree nor agree=3; Agree=4; Strongly agree=5.

^b^The N/A options are not given any weight.

**Table 6:** Faculty’s rating of students’ preparedness (N=12).

Faculty perceptions of students’ preparedness^a^		Unsure, n (%)	Not prepared at all, n (%)	Somewhat prepared, n (%)	Prepared, n (%)	Very prepared, n (%)	Mean (SD)
**How prepared are your matriculating medical students to follow weight loss guidelines to treat the following?**							
	Overweight patients	1 (−^b^)	0 (0)	8 (73)	3 (27)	0 (0)	2.27 (0.467)
	Stage I obese patients	3 (−)	0 (0)	6 (67)	3 (33)	0 (0)	2.33 (0.5)
	Stage II obese patients	3 (−)	1 (11)	5 (56)	3 (33)	0 (0)	2.22 (0.667)
	Stage III obese patients	3 (−)	1 (11)	7 (78)	1 (11)	0 (0)	2 (0.5)
	Patients with psychological or psychosocial issues	2 (−)	2 (20)	8 (80)	0 (0)	0 (0)	1.8 (0.422)
	Overweight or obese patients with weight-related comorbidities	1 (−)	0 (0)	5 (45)	6 (55)	0 (0)	2.55 (0.522)
	Overweight or obese patients with a history of failed weight loss attempts	4 (−)	4 (50)	4 (50)	0 (0)	0 (0)	1.5 (0.535)

^a^Likert rating: Unsure=N/A; Not prepared at all=1; Somewhat prepared=2; Prepared=3; Very prepared=4.

^b^The N/A options are not given any weight.

## Discussion

### Principal Findings

On the basis of the student and faculty responses, a medical school curriculum should stress on patient interviewing or counseling skills to address weight issues with all patients and associated weight-related problems. Specific areas of need in current medical school curricula include providing real or simulation-based opportunities for students to practice clinical skills while interacting with patients, such as discussing weight-related issues with patients in a patient-centered way, to increase quality and effectiveness of these interactions, and to decrease discomfort with these measures. Furthermore, students need experience with a variety of patients needing weight interventions, including those with extreme obesity and psychological problems. Simulations would also offer the opportunity to become comfortable with skills, such as bringing up the topic of weight or measuring waist circumference.

Currently, obesity-related topics are primarily integrated into traditional basic science and systems–oriented courses, from Biochemistry or Nutrition to Endocrinology and Cardiovascular Systems. This approach may not be the most effective way to teach students about this public health threat. Although most students were familiar with the topics, few reported case studies or clinical simulations that focused on obesity and developed the necessary patient interaction skills.

Faculty and students identified several similar deficits in obesity medicine training. Only around half of both faculty and students felt that students were prepared to follow weight loss treatment guidelines. Students were not confident of their interviewing and motivational skills, and faculty saw this as an important skill area to supplement. Both students and faculty saw a deficit in being able to help patients who have many failed weight loss attempts.

Faculty and students differed on their perception of how well prepared they are in obesity medicine in a few areas. Faculty rated students as being prepared to treat weight-related comorbidities more frequently than students did. Faculty believed that students were being appropriately prepared in the evaluation and assessment of patients with overweight or obesity, but students rated their preparation in some related skills low, such as measuring waist circumference.

Most students in round 1 reported interest in learning more about obesity and saw treatment as part of their role as a health care provider, including covering areas such as diet and lifestyle. In the follow-up round, however, students indicated that they see treating obesity as frequently secondary to “more important health problems.”

More emphasis is needed on evidence-based guidelines for treating all stages of excess weight, as well as developing and implementing long-term plans for patient weight loss. Motivational interviewing and other counseling techniques need to be incorporated more into the medical school experience, as both students and faculty agree that this is an area where there is room for improvement. Students overwhelmingly indicated interest in this topic and other topics related to patient communication.

All 3 lines of inquiry (literature review, medical student, and faculty feedback) support the inclusion of more training for students related to weight biases and the implications for patient care.

### Limitations

The sample size of our student and faculty populations was small, and therefore the findings could be seen as not necessarily representative of the populations as a whole. Although all attempts were made to expand our sampling through direct email recruitment, we were limited in our access to medical school contact information and therefore had to rely on convenience sampling of those users who made their contact information public, visited our site, and took our survey.

Analyzing the originating university of each participant as well as their current year or position, our study did include 2 distinct subsets of each population that could be seen as a representative sample of both students and faculty. The majority of student respondents were from Kansas University Medical School (11), but our sample covered a range of medical student years and therefore can be seen as representative of the medical school population as a whole. Additionally, our faculty sample had representatives from 12 different universities with no duplicates and therefore could be used as a representative sample of the medical school faculty population overall.

### Conclusions

Most students and faculty agreed that medical school curricula are preparing the students appropriately to discuss medical comorbidities and assess weight. A troubling finding was that the medical students surveyed did not feel *adequately prepared* to interview, assess, or treat a patient with overweight or obesity, particularly given that this is an audience with generally high confidence. Faculty should consider supplementing and changing curricula to address this concern. As medicine as a whole moves toward the realization that obesity is itself a health condition, not just a symptom or sign of other medical problems, these students will not be ready to assist their future patients unless changes are made in their training.

## Abbreviations

AAMC: Association of American Medical College
BMI: body mass index
